# Intracranial aneurysms in Ghanaian adults

**DOI:** 10.4314/gmj.v57i3.13

**Published:** 2023-09

**Authors:** Benjamin D Sarkodie, Bashiru B Jimah, Abdullah H Mohammed, Albert Akpalu, Edmund K Brakohiapa, Dorothea Anim, Benard O Botwe

**Affiliations:** 1 University of Ghana Medical School, Radiology, Accra, Ghana; 2 University of Cape Coast, School of Medical Sciences, Medical Imaging. Cape Coast, Ghana; 3 Korle Bu Teaching Hospital, Neurosurgery, Accra, Ghana; 4 Korle Bu Teaching Hospital, Internal Medicine, Accra, Ghana; 5 University of Ghana Medical School, Radiology, Accra, Ghana; 6 Korle Bu Teaching Hospital, Neurosurgery, Accra, Ghana; 7 University of Ghana, Radiography Department, Radiology, Accra, Ghana

**Keywords:** Intracranial, aneurysms, angiography, Ghanaian, adults

## Abstract

**Objective:**

To document the location, size, and multiplicity of intracranial aneurysms in Ghanaians who have undergone digital subtraction angiography (DSA) at a single centre in Accra, Ghana.

**Design:**

We conducted a retrospective observational review of the medical records of all patients diagnosed with intracranial aneurysms on DSA

**Setting:**

Patients' medical records at Euracare Advanced Diagnostic and Heart Centre were reviewed between March 2018 and March 2020.

**Participants:**

Thirty-one patients were identified with various intracranial aneurysms (IAs) within the study period. Patients' ages, sex, and types of IAs were extracted using a checklist and analysed using Microsoft Excel for Windows 2016.

**Interventions:**

None

**Main outcome measures:**

The prevalence of types and distribution of intracranial aneurysms.

**Results:**

The age range of the patients was 26-76 years, with a mean age of 45.5±14.3 years. The mean age of men and women with IA was 45.5 ±15.9 years and 46.7 51.3±12.9 years, respectively. The most common IAs were located in the posterior communicating artery (PCOM) at 54.8% (95%CI: 36.0, 72.7), followed by the anterior communicating (ACOM), which constituted 32.3% (95%CI: 16.7, 51.4). The majority, 89.2% (33/37) of these aneurysms were less than 7mm in diameter. Single aneurysms were present in 25 (80.6%).

**Conclusion:**

The most common IAs were found in the PCOM and ACOM, and IAs tend to rupture at a younger age and smaller size among the Ghanaian adults examined. Early detection and treatment of IAs less than 7mm in diameter is recommended.

**Funding:**

None declared

## Introduction

Intracranial aneurysms (IAs)contribute significantly to morbidity and mortality in the adult population world-wide.^1^,^2^,^3^,^4^In a systematic review, the global prevalence of IAs was between 3.7% and 6.0%.^1^A study in Ghana reported 16.6% incidence of IA among hypertensives and 8.5% in normotensives from an autopsy data.^2^IA is associated with genetic diseases such as autosomal dominant polycystic kidney disease (ADPKD), neurofibromatosis, alpha-1antitrypsin deficiency, and tends to run in families with a prevalence of 7% to 20% in first- or second-degree relative of patients who have previously suffered from SAH.^3^,^4^

Four main types of intracranial aneurysms are commonly encountered: fusiform, saccular, dissecting, and mycotic, with the saccular type being the most common. Over eighty-five per cent of saccular aneurysms tend to occur in the circle of Willis, with 30% said to be multiple.^3^

Many IAs are found incidentally during imaging, while some present with clinical symptoms such as headaches, photophobia, cranial nerve disturbances, confusion, etc. Up to 15% of unruptured aneurysms are symptomatic.^8^,^9^

Subarachnoid haemorrhage is a dreaded clinical consequence of intracranial aneurysmal rupture.^9^ Sentinel headache, which manifests as severe thunderbolt headache occurring days before aneurysmal rupture, may be seen in 10-43% of subarachnoid haemorrhage patients^13^ and may carry a higher risk of re-rupture.^14^ Sudden loss of consciousness can also be a point of sentinel bleed or true aneurysmal rupture.

The three main imaging modalities used in the diagnosis of intracranial aneurysms are computed tomography angiography (CTA), Magnetic Resonance Angiography (MRA), and Digital Subtraction Angiography (DSA).^15^-^18^ComputedTomography Angiography (CTA) is a commonly used modality for imaging and screening for intra-cranial aneurysms because it is readily available and non-invasive. Evidence from meta-analysis shows a relatively acceptable level of sensitivity [95% (95% CI: 92-97%)] and specificity [98.9% (95% CI: 91.5-99.99%)] for detecting 7 mm aneurysms.^17^ DSA is the gold standard imaging method for intracranial aneurysms. Using techniques like 3D rotation, even very small aneurysms can be identified.^15^ Another advantage of DSA is the ability to define relationships between the smaller branches and perforators near the aneurysm and to help define an aneurysm from an infundibulum. DSA is a relatively safe procedure with minor complications and less than 1% risk of arterial dissection, acute ischemic stroke and bleeding.^16^

In Ghana, little is reported on intracranial aneurysms in the adult population. The purpose of this study was to document the location, size, and multiplicity of IA in Ghanaians who have undergone DSA at the Euracare Advanced Diagnostics and Heart Centre in the Greater Accra region of Ghana.

## Methods

The study was a retrospective cross-sectional study of patients who presented at the Euracare Advanced Diagnostics and Heart Centre for diagnostic and interventional digital subtraction angiography (DSA) between March 2018 and March 2020. Data confidentiality, patient anonymity, and privacy were ensured by the lead investigator sorting information in a password-protected computer. The Cape Coast Teaching Hospital Ethical Review Committee approved the study with CCTHERC/EC/2022/059 reference number.

Between March 2018 and March 2020, 31 patients were diagnosed with intracranial aneurysms during cerebral digital subtraction angiography. The medical records of all the patients were reviewed. A checklist was developed to extract data from imaging records, and documented variables included age, gender, and location of intracranial aneurysms, rupture status, and diameter of intracranial aneurysms.

Extracted data were analysed with descriptive statistics such as frequency and percentages. The diameter of each aneurysm was recorded via the imaging assessment. The combined sizes were computed to generate the mean size and standard deviation for an aneurysm type and category (i.e. unruptured and ruptured The association was assessed using the Fischer exact test from Statistical Package of Social Science (IBM SPSS version 21). Charts were generated using Microsoft Excel for Windows 2016.

All patients seen at the facility had DSA using the Seldinger technique to site a 6F or 7F sheath after ultrasound-guided puncture of the right common femoral artery using a 0.035' hydrophilic guidewire and a DAV catheter (and sometimes HN5 catheter) under fluoroscopic guidance, the angiograms of the internal carotid and vertebral arteries were obtained. Documented aneurysms were characterised by size, location, shape, and cause. The aneurysm sizes were classified into smaller than 7 mm in diameter and larger than 7 mm in diameter.

The aneurysm locations were classified as follows: anterior communicating artery (AComA), middle cerebral artery (MCA), posterior communicating artery (PComA), anterior cerebral artery (ACA), internal carotid artery (ICA) and vertebro-basilar system (VB). Patients were categorised according to their presentation and CT scan findings into SAH ruptured and unruptured groups. The aneurysms were also classified as Saccular or berry aneurysms, dissecting fusiform based on shape.

## Results

A total of 37 lesions were noted in 31 patients. Male to female ratio was 1:1.8. The age range of the patients was 26-76 years, with a mean age of 45.5±14.3 years. The mean ages of men and women were 45.5 ±15.9 years and 46.7 51.3±12.9 years, respectively. Thirteen (41.9%) of the patients were aged 21-40 years (Table 1).

**Table 1:** Age and sex distribution of patients with intracranial anuerysms

Gender	Age range (years)			Total
	21-40	41-60	Above 60	
	N (%)	N (%)	N (%)	N (%)
Male	5 (45.5)	2(18.2)	4 (36.4)	11 (100.0)
Female	8 (40.0)	8(40.0)	4 (20.0)	20 (100.0)
Total	13 (41.9)	10(32.3)	8 (25.8)	31 (100.0)

At a confidence level of 95%, IAs in the posterior communicating artery (PCOM) were noted in 17 patients (54.8%), the other locations are described in Table 2. Saccular aneurysms accounted for thirty-six cases, while a single fusiform cavernous aneurysm was noted. Ruptured aneurysms were present in 27(72.9%) intracranial aneurysms, all of which were saccular, while 10(27.1%) were unruptured. No dissecting aneurysms were detected in this study. Patients with internal carotid artery (ICA) aneurysms had the largest mean size (13.4 mm) of an unruptured aneurysm (Table 2).

**Table 2:** Aneurysmal characteristics of the patients

Location*	Ruptured	Unruptured			All patient (n=31)	
	n=27	Mean size (mm)	n=10	Mean size (mm)	N=37	% (95%CI)
**Anterior circulation (n=20):**						
**ICA**	0	-	4	13.4	4	12.9 (3.6, 29.8)
**ACA**	0	-	4	4.0	4	12.9 (3.6, 29.8)
**ACOM**	10	4.8	0	-	10	32.3 (16.7, 51.4)
**MCA**	1	4.5#	1	3.0#	2	6.5 (0.8, 21.4)
**Posterior circulation (n=17):**						
**PCOM**	16	4.2	1	2.8#	17	54.8 (36.0, 72.7)
**VBS**	0	-	0	-	-	0

* ACOM-anterior communicating artery; MCA-middle cerebral artery; PCOM-posterior communicating artery; ACA-anterior cerebral artery; ICA- internal carotid artery; and VBS -vertebro-basilar system

# Actual size of aneurysm diameter

**Figure 1:**
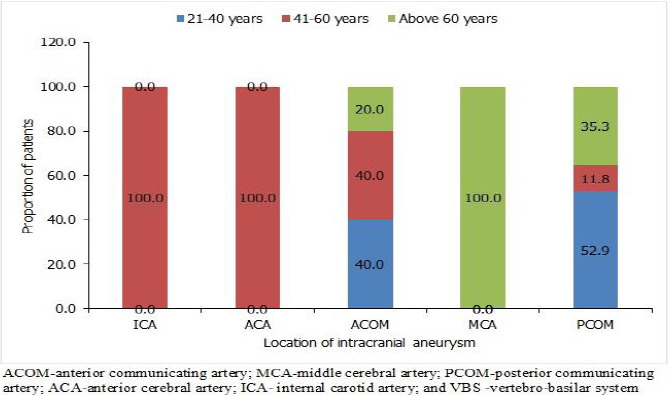
Location of intracranial aneurysms by age group of patients

A greater proportion, 89.2% (33/37) of these aneurysms were less than 7mm in diameter. ICA and MCA aneurysms mostly affected females, while males were much affected by ACA and PCOM aneurysms. As shown in Figure 1, all intracranial aneurysms in ICA and ACA were among patients aged 41-60 years, while the intracranial aneurysms in MCA were found among patients above 60 years.

Most patients had a single aneurysm, 25 (80.6%), while 6 (19.4%) had multiple aneurysms. Multiple aneurysms of the intracranial arteries mostly affect males (45.5%) and patients above 60 years (50%). Presentation of multiple aneurysms of intracranial arteries was significantly associated with gender (p=0.013) and age (p=0.009) of patients (Table 3).

**Table 3:** Proportion of multiple aneurysms among patients

Characteristic	Single aneurysm	Multiple aneurysms	Fischer exact test
	n (%)	n (% )	p-value
Total patients	25 (80.6)	6 (19.4)	-
**Gender**			0.013*
Male	6 (54.5)	5(45.5)	
Female	19 (95.0)	1 (5.0)	
**Age (years)**			0.009*
21-40	13(100.0)	0 (0.0)	
41-60	8 (80.0)	2(20.0)	
Above60	4 (50.0)	4 (50.0)	

## Discussion

The design of the study being retrospective and the small study population limited this study. Therefore, the findings cannot be generalised to the entire population of Accra. Although many studies have demonstrated aneurysms in older populations, the current study found aneurysms in the younger populations with a mean age of 45.5 years, mostly between the ages of 21 to 40 years. The finding is consistent with previous studies from Sub-Saharan Africa.^20^,^21^Swartbooi et al. reported a mean age of 45 years in Bloemfontein, While Julius et al. observed a mean of 50.9 years in Nairobi.^20^,^21^ However, studies in Asia and Europe found that the patients were about a decade older.^22^,^23^,^24^ This can be attributed to the younger age of the African study population.

Intracranial aneurysms have a high prevalence in females.^25^ This study had a female: male ratio of 1.8: 1, which is similar to the published international series.^25^However, some studies have shown an even higher prevalence of 3: 1 in favour of women.^26^,^27^

There are regional variations in the incidence of SAH, a complication of untreated intracranial aneurysms, with the incidence higher amongst Finnish and Japanese populations.^25^ The gender disparity increases with age, with the incidence in females approaching three times that of males^28^ The current study shows a higher incidence of aneurysmal rupture in females with SAH, 1.6 times more common in women. Juvela et al. suggests that active cigarette smoking history and female gender are the only independent risk factors for aneurysm growth. 29 Also, the link between low oestrogen levels and the likely alteration of arterial integrity, gender differences in vascular geometry, and wall shear stress (WSS) may increase the risk of SAH in females.^30^-^37^

Multiple IAs were seen in 19.4% of these patients and predominantly in males. This finding is consistent with the 17% prevalence reported from Hong Kong and 17.7% in Japan.^22^,^38^ However, their findings showed a female predominance. The frequency of IAs varied considerably according to the anatomic location.^20^,^21^,^23^,^39^In the present study, the incidence of anterior and posterior circulation aneurysms was 54% and 46%, respectively. Although the findings of this study could not explain the possible reasons for the higher incidence in the anterior segments of the cerebral circulation, previous studies have linked cerebral aneurysms with cigarette smoking, hypertension, previous personal and family history of aneurysmal subarachnoid haemorrhage, and excessive alcohol consumption.^20^,^21^,^23^,^40^,^41^ The most common aneurysm location in this study was in the posterior communicating artery, 46%, which sharply contrasts several others which found the ACOM as the most common site.^20^,^21^,^23^,^39^However, this finding is consistent with a study by Lai et al. who found the PCOM to be the commonest site for intracranial aneurysms.^22^ The commonest intracranial aneurysm is saccular, whereas dissecting, fusiform, infectious, traumatic, and mycotic aneurysms are rare.^41^ This is consistent with the findings in the current study, where 97.3% of the aneurysms were saccular.

In the current study, no intracranial aneurysm was seen in vertebro-basilar arteries, confirming suggestions by other publications that vertebro-basilar aneurysms are rare.^42^ A similar study by Peluso et al. found less than 0.5% of intracranial aneurysms in the vertebro-basilar arteries of the posterior circulation.^42^ Similarly, Lai et al. found 8.3% of intracranial aneurysms involving the basilar artery (5.6%) and vertebral artery(2.7%) among patients in China.^22^ Vertebro-basilar junction (VBJ) fenestration is strongly associated with and directly correlates with aneurysm formation, with a reported incidence of 35%.^43^ The continental variation in VBJ aneurysms may be due to differences in the occurrence of fenestrations.

It has been reported that ACOM and multiple aneurysms are significantly associated with an increased risk of intracranial bleeding and poor modified Fisher grade.^44^In the International Study of Unruptured Intracranial Aneurysms(ISULA), the authors reported that patients with no history of SAH and asymptomatic anterior circulation aneurysm <7mm do not require treatment as the risk of rupture was insignificant.^45^,^46^ In this study, the mean ruptured aneurysm size was less than 4.5mm, in line with the South African Study.^20^ Similar to the International Subarachnoid Aneurysm Trial (ISAT), the findings of the index study contradict those of the International Study of Unruptured Intracranial Aneurysms trial, which revealed that anterior circulation aneurysms less than 7 mm have a 3%chance of rupturing at five years.^47^ An earlier study conducted by Juvela et al. 30 found that 67% of the ruptured aneurysms in their series were less than 6 mm, further validating our findings. Juvela and his colleagues concluded that an unruptured aneurysm should be operated on regardless of size—the caveat is that the intervention/surgery must be technically possible.

## Conclusion

This study shows that ruptured aneurysm sizes are smaller than reported in most international series but similar to most single-centre studies. This study also shows the predominance of posterior communicating artery aneurysms, which tend to rupture at a younger age and the preponderance of multiple aneurysms in males. Further studies are required to assess the sex disparity and other associated risk factors for ruptured aneurysms.
